# Clustering in multiple long-term conditions: methodological and translational challenges and solutions

**DOI:** 10.1177/01410768251395387

**Published:** 2025-12-03

**Authors:** Thomas Beaney, Alex Dregan, Myer Glickman, Pauline Mountain, Kamlesh Khunti, Hajira Dambha-Miller

**Affiliations:** 1 The George Institute for Global Health, Imperial College London, London, UK; 2 Department of Psychological Medicine, King’s College London, London, UK; 3 Office for National Statistics, Newport, UK; 4 Cross-NIHR-Collaboration in MLTC, Leicester, UK; 5 University of Leicester, Diabetes Research Centre, Leicester General Hospital, Leicester, UK; 6 Primary Care Research Centre, University of Southampton, Southampton, UK

Clustering, the process of grouping long-term conditions (LTCs) or people based on shared characteristics, disease patterns, risk factors or clinical trajectories, has become a prominent focus of research on multiple long-term conditions (MLTC).^1,2^ Its potential for delivering personalised care tailored to specific patient phenotypes is significant, particularly as the burden of MLTC continues to rise globally.^3,4^ However, its promises remain largely unfulfilled in clinical practice due to methodological and translational challenges

## How clustering works and its targets in MLTC research

Clustering refers to a set of analytical methods that automatically group similar items together based on shared features. In the context of MLTC, these items can be either people or conditions. Algorithms compare hundreds of characteristics – such as age, diagnoses, test results or healthcare use – to identify natural groupings that occur in the data, without researchers specifying them in advance. For example, one cluster might contain people with diabetes, obesity and hypertension who frequently attend the hospital, while another may include younger adults with mental health conditions and chronic pain but lower healthcare use. Each cluster, therefore, represents a group of people with broadly similar health profiles and care needs. The usefulness of clustering lies in its ability to simplify complexity. Rather than treating every patient with multiple conditions as unique, clustering helps clinicians and policymakers understand which patterns commonly occur together, how these groups differ in prognosis and what types of interventions may work best for each group. In practice, this can support risk stratification, inform targeted prevention programmes and guide service design – such as developing integrated clinics for people who fall within high-risk clusters.

In MLTC research, clustering usually aims to identify groups of LTCs, based on their co-occurrence, or clusters of people, based on similarity in their diseases, care needs or healthcare outcomes (also called patient segmentation).^2,5^ Of studies clustering people, some do so directly based on a measure of similarity,^5–7^ while others first cluster LTCs and subsequently assign individuals according to their conditions.^8,9^ The target of clustering should reflect its intended application. For example, LTC clusters inform shared biological mechanisms and therapeutic drug discovery,^1,10^ whereas people clusters provide more direct clinical insights, such as for risk stratification or to inform services designed around care of specific clusters.^5,9^

## Challenges in applying clustering to MLTC

While clustering holds great promise for advancing the understanding and management of MLTC, its translation into clinical practice faces several challenges. These include methodological limitations – such as narrow condition selection and restricted data inputs – as well as issues related to data quality, bias and the absence of meaningful patient and carer involvement. Addressing these challenges requires not only technical advances, including the use of artificial intelligence and longitudinal data, but also improvements in data linkage, inclusivity and research co-design. The following sections outline key challenges and opportunities to overcome them.

## Choice of LTCs

A weakness of many clustering approaches in MLTC research is the focus on common conditions only.^11^ Although individually uncommon, rare diseases are collectively common, affecting 1 in 17 people in the United Kingdom.^12^ Rare conditions can have a disproportionate impact on people living with them, often compounded by a lack of knowledge of the disorder by health professionals, which in turn can negatively affect mental health.^13^ People with rare conditions may also experience complex interactions between their rare and more prevalent diseases that require differing treatment approaches. For example, effective management of diabetes or cardiovascular disease in cystic fibrosis must consider the unique challenges posed by the combination.^14^ To improve clinical applicability, clustering approaches should include rare conditions, to better capture the diversity of people’s experiences.

## Broadening data inputs

Another weakness is a focus on the presence or absence of LTCs as binary health states. Disease severity (such as HbA1c or blood pressure in diabetes), symptoms and impact (such as functional impairment after stroke) are rarely accounted for but substantially influence care needs. Beyond clinical data, clustering often overlooks other crucial factors affecting health. Most studies focus on demographics, biomarkers, genetic predispositions and disease-specific characteristics, but MLTC is influenced by a complex array of social, psychological and lifestyle factors (such as diet, physical activity, smoking and alcohol consumption) that affect disease progression, care needs and health outcomes.^15^ For example, socioeconomic status, physical and mental well-being and access to healthcare play a major role in the management of conditions such as diabetes and chronic obstructive pulmonary disease.^16,17^ The predominant focus on biological phenotypical factors fails to capture the real-world complex care needs of people with MLTC, limiting the practical value of clusters for personalised patient care.

Limited availability of such data in routinely collected health records further contributes to this challenge, made more difficult by a plethora of technical and governance barriers. Despite some examples of regional success, national mechanisms for linkage of NHS, voluntary sector and local authority social care records are lacking. Neither is there yet a single coding or classification system capable of facilitating streamlined data analysis across all the record systems (e.g. GP, hospital, social care), conceptual domains (e.g. diagnosis, treatment, functioning) and patient-reported data involved. Realising the potential of clustering requires greater linkages with non-health-related data, including social care and patient-reported information.^18^

## Opportunities using AI

Artificial intelligence (AI) could help address some of these limitations. Machine learning algorithms enable the analysis of large, complex datasets, including many data inputs, allowing for the creation of clusters that better reflect the range of factors affecting people’s health.^19^ Similarly, data limitations may in future be partly addressed by natural language processing (NLP) methods capable of extracting information from the richer unstructured data which make up the bulk of information entered during clinical encounters.^20,21^ However, several challenges are associated with AI. First, incomplete or biased data can affect the reliability and fairness of AI algorithms, and it remains unclear whether the use of NLP with unstructured data can address this or may exacerbate bias.^22,23^ Second, the transparency of AI models and risk of bias remains a concern. Given the potential for AI-based clustering to perpetuate existing biases in healthcare, assessment of fairness must be considered in algorithm design, including assessment of equitable performance across population groups. Clinicians and patients also require clear explanations of how AI algorithms reach their conclusions to promote trust and support their safe and effective implementation in clinical practice, an area in which research is evolving.

## Incorporating patient and informal carer priorities

Another weakness limiting the clinical impact of MLTC clustering is insufficient consideration of patient and informal carer opinions on what matters to them regarding disease burden, interactions and associations with adverse outcomes. This is vital given that the experiences of people living with MLTCs are often misunderstood by healthcare staff,^24^ and patient-centred care often falls short due to this mismatch.^25^ Furthermore, informal caregiver (such as family, friends or neighbours) experiences are often overlooked, and carer priorities may differ from those of patients.^26^ Patients and their carers must be involved throughout the research process, from selecting relevant inputs and outcomes, interpreting clusters and co-designing subsequent interventions, to ensure that clustering genuinely informs patient-centred care.

## Adopting new methodologies

Advances in clustering methods may enable greater clinical impact. Unsupervised clustering relies on either patient characteristics (such as demographics and clinical conditions) or health outcomes (such as healthcare utilisation or incidence of a new condition). In the first case, people within the same cluster may appear similar, but their outcomes will diverge over time, limiting practical relevance to understanding future care needs. In the second case, people with similar outcomes may have disparate conditions, making it challenging to understand the mechanisms underlying the clustering and tailor management to a given cluster. Supervised methods, including semi-supervised or outcome-aware clustering methods, informed by future clinical events such as hospital admissions or mortality,^27,28^ are promising methodological approaches which could enhance the clinical relevance of MLTC clusters by balancing current characteristics and future outcomes.^19,29^

MLTC is a dynamic process that evolves over time, but most clustering studies rely on cross-sectional data, which provide a snapshot of people’s characteristics at a single point in time, without considering the order in which they developed.^30^ Longitudinal clustering using data sources which track people over time, either prospectively or retrospectively, can provide a deeper understanding of how people transition across clusters as they age, and how their experiences and needs change.^6^ AI algorithms, such as transformers, can more accurately predict patient outcomes than methods relying only on static information.^31–33^ They can also be integrated into clustering pipelines to produce clusters that better anticipate future care needs and inform long-term care.^34^

## Implementation: embedding translation from the start

While AI offers promise, a translational gap remains between clustering research and its implementation into clinical practice, without clear examples of data-driven clustering informing changes to models of care. One explanation is the methodological focus on the clusters themselves, rather than the practical challenges of implementing cluster-based interventions. Current clustering research is often exploratory, without a clear path to clinical integration, which limits actionable findings. Addressing this requires the objectives to be explicitly defined from the outset, guiding the selection of data inputs, choice of conditions and validation. While validation strategies such as assessing stability and clinical plausibility are important,^35^ demonstrating the utility of clusters in the real world provides the most impactful validation. Without such evidence of clinical effectiveness, healthcare providers may be reluctant to adopt clustering-informed care models.

Co-designing implementation and evaluation strategies with patients, clinicians and service managers should be considered from the outset. Practical considerations include the feasibility of changes to clinical workflows, including the integration of new tools and the development of training for healthcare professionals and implementation of different models of care. Rigorous evaluation also requires access to high-quality clinical data, which may be hindered by organisational barriers to data sharing and linkage. Without appropriate resources and infrastructure to pilot and evaluate them, clustering will remain a theoretical construct rather than a practical tool for improving care.

## Recommendations

1. Expand clustering data inputs, integrating clinical, lifestyle, psycho-social and patient-reported factors.
2. Enable integration and linkage of large population-level data sets.
3. Include rare conditions in clustering analyses.
4. Embed patient and informal carer perspectives throughout all stages of the research.
5. Develop semi-supervised, outcome-aware clustering algorithms that produce clusters with similar characteristics and prognostic information.
6. Advance methodologies for generating clusters incorporating longitudinal information.
7. Plan implementation and evaluation pathways from the outset of research design.

## Conclusion

Clustering has considerable potential to improve MLTC management through personalised and targeted care. Realising this potential requires overcoming the methodological limitations, by reducing reliance on biological factors, including rare conditions and actively incorporating patient and carer perspectives. While AI offers opportunities to address some of these challenges, including generation of outcome-aware and longitudinal clusters, issues surrounding data quality, algorithm transparency and clinical implementation must be addressed. To bridge the gap between research and clinical application, future research should prioritise developing more inclusive, longitudinal and person-centred clustering models and overcoming the real-world barriers to their effective use.

## Declarations

**Ethical considerations:** Not applicable.

**Consent to participate:** Not applicable.

**Consent for publication:** Not applicable.

**Provenance:** Not commissioned; peer-reviewed by Ansu Basu.
